# Safety and Efficacy of Upadacitinib in Crohn’s Disease: An Updated Systematic Review

**DOI:** 10.7759/cureus.70125

**Published:** 2024-09-24

**Authors:** Aliu O Olatunji, Muhammad Maqbool, Muhammad Ali Abid, Karthik Sai Makineni, Mohammed Khaleel I.KH. Almadhoun, Hamdah B Meer, Fazeela Ansari, Alma M Alfakhori, Adees W Bedros, Nasreen Banu, Syed Faqeer Hussain Bokhari

**Affiliations:** 1 Medical Microbiology, The University of Toledo, Toledo, USA; 2 Internal Medicine, Shaheed Mohtarma Benazir Bhutto Medical College Lyari, Karachi, PAK; 3 Medicine, King Edward Medical University, Lahore, PAK; 4 Medicine, Sri Ramaswamy Memorial (SRM) Medical College Hospital and Research Centre, Chennai, IND; 5 Medicine and Surgery, Mutah University, Mu'tah, JOR; 6 Medicine and Surgery, Dubai Medical College for Girls, Dubai, ARE; 7 Internal Medicine, School of Medicine, The University of Jordan, Amman, JOR; 8 Medicine, Shadan Institute of Medical Sciences College, Hyderabad, IND; 9 Surgery, King Edward Medical University, Lahore, PAK

**Keywords:** crohns disease, efficacy, inflammatory bowel disease, jak inhibitor, systematic review, ulcerative colitis, upadacitinib

## Abstract

Crohn’s disease (CD) is a chronic inflammatory bowel disease that significantly impacts patient quality of life. This systematic review evaluates the safety and efficacy of upadacitinib, a selective Janus kinase (JAK) inhibitor, in the treatment of CD. A comprehensive literature search was conducted across multiple databases, yielding seven studies published between 2020 and 2024, encompassing 1,481 patients. The review includes randomized controlled trials, post hoc analyses of phase 3 trials, and observational studies. Findings consistently demonstrate upadacitinib’s superiority over placebo in inducing and maintaining clinical remission, achieving endoscopic response, and normalizing inflammatory markers. Notably, upadacitinib showed rapid symptom relief, with clinical remission observed as early as five to six days after treatment initiation. Efficacy was observed across various patient populations, including those with prior biologic failure. Long-term studies indicated sustained clinical and endoscopic improvements, with remission rates maintained for up to 30 months. Upadacitinib also demonstrated effectiveness in real-world, treatment-refractory cohorts. Safety profiles were generally consistent with those of other JAK inhibitors. Common adverse events included infections, particularly herpes zoster, and laboratory abnormalities such as neutropenia and elevated creatine kinase. Serious adverse events were infrequent, although careful monitoring is warranted. This review suggests that upadacitinib is a promising treatment option for moderate to severe CD, offering rapid and sustained efficacy with an acceptable safety profile.

## Introduction and background

Crohn’s disease (CD) is a chronic, relapsing inflammatory bowel disease (IBD) characterized by transmural inflammation that can affect any part of the gastrointestinal tract. The pathophysiology of CD is complex, involving a dysregulated immune response to intestinal microbiota in genetically susceptible individuals, leading to chronic inflammation and tissue damage [1]. From 1990 to 2019, IBD has demonstrated a notable global burden, with 405,000 new cases and 41,000 deaths reported in 2019. The global age-standardized incidence rate was 4.97 per 100,000 person-years, with a slight decline observed over recent decades [2]. High-income regions, such as North America and Western Europe, have experienced stable or decreasing incidence rates, while lower-income regions, including parts of Asia and Latin America, have seen a rise in incidence. Despite these regional differences, the overall global burden of IBD remains significant, with substantial variations in incidence, mortality, and disability-adjusted life years across different sociodemographic and geographic contexts [2]. The impact of CD on patient quality of life is significant, often resulting in recurrent hospitalizations, surgeries, and substantial psychosocial burdens.

The current treatment landscape for CD encompasses a range of therapeutic options, including aminosalicylates, corticosteroids, immunomodulators (e.g., azathioprine and methotrexate), and biologic agents such as anti-tumor necrosis factor (anti-TNF) therapies [3]. While these treatments have shown efficacy in inducing and maintaining remission, they are associated with limitations. Corticosteroids, while effective for short-term use, are not suitable for long-term management due to their side effect profile [4]. Immunomodulators can be slow-acting and may increase the risk of infections and malignancies [5]. Biologic therapies, particularly anti-TNF agents, have revolutionized CD treatment but are associated with loss of response over time and potential adverse events [6]. These limitations underscore the need for new therapeutic options that can provide rapid, sustained efficacy with a favorable safety profile.

Upadacitinib, a selective Janus kinase (JAK) inhibitor, has emerged as a promising candidate for the treatment of CD [7]. JAK inhibitors represent a novel class of small molecule drugs that target the JAK-signal transducer and activator of transcription (STAT) signaling pathway, which plays a crucial role in immune cell activation and inflammatory processes. Specifically, upadacitinib preferentially inhibits JAK1, a key mediator in the signaling of multiple pro-inflammatory cytokines implicated in the pathogenesis of CD, including IL-6, IL-7, IL-15, and interferon-γ [8-10]. The rationale for investigating upadacitinib in CD stems from its demonstrated efficacy in other inflammatory conditions. Upadacitinib has shown significant clinical benefits in rheumatoid arthritis, leading to its approval by regulatory agencies for this indication [11]. Moreover, promising results have been observed in clinical trials for ulcerative colitis (UC), another form of IBD [12]. The oral administration of upadacitinib offers a potential advantage over injectable biologic therapies, potentially improving patient adherence and quality of life [13].

The potential benefits of upadacitinib in CD are multifaceted. By modulating the JAK-STAT pathway, upadacitinib may simultaneously target multiple inflammatory mediators involved in CD pathogenesis, potentially offering a more comprehensive approach to disease control. Additionally, the rapid onset of action observed with JAK inhibitors in other inflammatory conditions suggests the possibility of achieving quicker symptom relief in CD patients. Furthermore, the small molecule nature of upadacitinib may allow for better tissue penetration compared to larger biologic agents, potentially enhancing its efficacy in treating transmural inflammation characteristic of CD [14]. The primary objective of this systematic review is to assess the efficacy of upadacitinib in the treatment of CD. This assessment will encompass various clinical outcomes, including induction and maintenance of clinical remission, endoscopic improvement, and changes in inflammatory biomarkers. Additionally, this review aims to evaluate the safety and tolerability profile of upadacitinib in CD patients, considering both short-term and long-term treatment durations.

By comprehensively analyzing the available evidence on upadacitinib in CD, this review seeks to provide clinicians and researchers with a critical appraisal of its potential role in the therapeutic armamentarium for CD. The findings may help inform clinical decision-making, guide future research directions, and ultimately contribute to improving the management of patients with this challenging condition.

## Review

Materials and methods

This narrative systematic review was conducted to comprehensively evaluate the efficacy of upadacitinib in the treatment of CD. The review process adhered to the Preferred Reporting Items for Systematic Reviews and Meta-Analyses (PRISMA) 2020 guidelines to ensure a thorough and transparent approach to literature synthesis [15].

Search Strategy

A comprehensive literature search was performed using multiple electronic databases, including MEDLINE (via PubMed), Scopus, Cochrane Central Register of Controlled Trials (CENTRAL), and Web of Science. The search period spanned from the inception of each database to May 31, 2024. The search string used was “(upadacitinib AND Crohn’s disease)”. To ensure comprehensive coverage, we also conducted a manual search of reference lists from pertinent reviews and included studies to identify any additional relevant publications that may have been overlooked in the initial database search.

Eligibility Criteria

Studies were deemed eligible for inclusion if they met specific criteria. The population of interest consisted of adults (aged 18 years or older) with a confirmed diagnosis of CD, irrespective of disease duration or treatment history. The focus of the intervention was upadacitinib, either as monotherapy or in combination with standard CD treatments. Comparators included placebo, no treatment, or other active treatments for CD. The primary outcomes of interest were the induction and maintenance of clinical remission, as well as endoscopic improvement or remission. Secondary outcomes included changes in inflammatory biomarkers, health-related quality of life, adverse events, and treatment discontinuation rates. We prioritized randomized controlled trials (RCTs) for inclusion but also considered observational studies, particularly for insights into long-term efficacy and safety. Gray literature, case reports, and case series were excluded. Studies not in the English language were also excluded..

Study Selection Process

The study selection process was carried out independently by two reviewers. Initially, they screened the titles and abstracts of all retrieved records to exclude clearly irrelevant studies. Subsequently, full-text articles of potentially eligible studies were obtained and reviewed independently by both reviewers. Any disagreements regarding study inclusion were resolved through discussion, with a third reviewer consulted when necessary to reach a consensus.

Data Extraction

Data extraction was performed using a standardized form, which was pilot-tested before full implementation. Two reviewers independently extracted relevant information from each included study. The extracted data encompassed study characteristics (such as author, year of publication, country, study design, and duration), participant demographics (including age, sex, disease duration, and prior treatment history), intervention details (upadacitinib dose and duration of treatment), and outcome measures as defined in the eligibility criteria.

Data Analysis

Given the narrative nature of this review, we focused on a qualitative synthesis of the findings rather than a statistical meta-analysis. The results from individual studies were summarized and integrated to provide a comprehensive overview of the efficacy of upadacitinib in CD. We paid particular attention to patterns across studies, consistencies and inconsistencies in findings, and potential factors that might explain variations in outcomes.

Results

Study Selection

**Figure 1:**
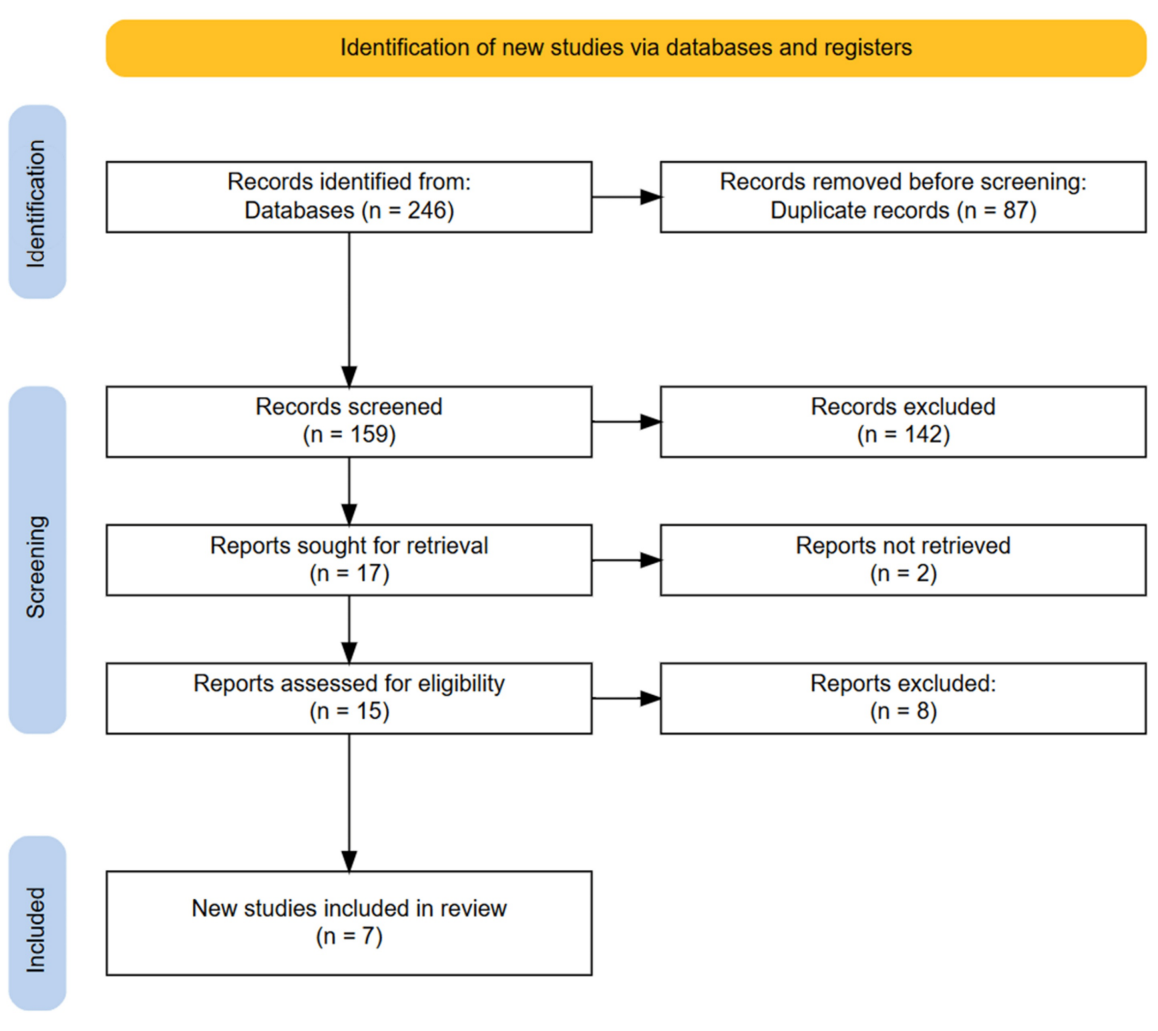
PRISMA diagram showcasing the study selection process PRISMA, Preferred Reporting Items for Systematic Reviews and Meta-Analyses

An initial search of the databases yielded 246 articles. After removing 87 duplicate entries, we screened the titles and abstracts of the remaining 159 publications. From this initial screening, 17 studies appeared potentially relevant and were subjected to a full-text review to assess eligibility. After a detailed evaluation, seven articles met our predefined inclusion criteria and were included in the final analysis. A manual review of the reference lists from the selected articles did not reveal any additional eligible studies. The entire selection process is depicted in the PRISMA flowchart (Figure 1).

Study Characteristics

This systematic review included seven studies published between 2020 and 2024, encompassing a total of 1,481 patients with CD. The studies varied in design, including RCTs, post hoc analyses of phase 3 trials, and observational studies. The largest studies were post hoc analyses by Colombel et al. (2024), Peyrin-Biroulet et al. (2024), and Loftus et al. (2023) [16-18]. They pooled data from two phase 3 induction trials (U-EXCEL and U-EXCEED) and one maintenance trial (U-ENDURE), involving 1,021 patients. Colombel et al. (2024) evaluated the efficacy of upadacitinib 45 mg once daily compared to placebo in adults aged 18-75 years with moderately to severely active CD [16]. Peyrin-Biroulet et al. (2024) performed another post hoc analysis of the same phase 3 trials, focusing on the efficacy of upadacitinib in patients with and without prior biologic failure [17]. This study included 1,021 patients, with 733 having prior biologic failure. Loftus et al. (2023) also analyzed the pooled data from the phase 3 trials, evaluating the efficacy and safety of upadacitinib for both induction (45 mg once daily) and maintenance (15 mg or 30 mg once daily) therapy in 1,021 patients [18].

Elford et al. (2024) conducted a retrospective, multicenter cohort study in the United Kingdom, including 93 patients with a median age of 36 years and a median disease duration of 12 years [19]. This real-world study assessed the effectiveness of upadacitinib in a highly treatment-refractory population, with 98% of patients having prior exposure to anti-TNF therapy. Friedberg et al. (2023) conducted a prospective cohort study in the United States, including 105 patients with UC or CD, of whom 40 had CD [20]. This study assessed the efficacy of upadacitinib (45 mg daily) for eight weeks in patients previously treated with biologics. D'Haens et al. (2022) reported on a phase 2, multicenter, open-label extension study of 107 patients who had completed the 52-week CELEST study [21]. Patients received either 15 mg or 30 mg of an extended-release formulation of upadacitinib once daily. The earliest included study was by Sandborn et al. (2020), a randomized, double-blind, placebo-controlled, dose-ranging phase 2 study involving 220 patients [22]. This study evaluated various dosing regimens of upadacitinib for both induction (3 mg, 6 mg, 12 mg, or 24 mg twice daily, or 24 mg once daily for 16 weeks) and maintenance (3 mg, 6 mg, or 12 mg twice daily, or 24 mg once daily for 36 weeks) therapy.

The primary efficacy outcomes across studies included clinical remission (often defined by a Crohn’s Disease Activity Index (CDAI) score <150), endoscopic response or remission, and changes in inflammatory biomarkers such as CRP and fecal calprotectin. Safety outcomes were reported in most studies, with a focus on adverse events, serious adverse events, and specific events of interest such as infections, particularly herpes zoster. The patient populations across studies were generally similar, consisting of adults with moderate to severe CD, many of whom had prior exposure to biologic therapies. The duration of follow-up ranged from eight weeks in the shorter-term studies to up to 30 months in the open-label extension study by D'Haens et al. [21]. Overall, these studies provide a comprehensive evaluation of upadacitinib in CD, including data from rigorous RCTs, real-world observational studies, and longer-term extension studies, allowing for a thorough assessment of its efficacy and safety profile (Table 1).

**Table 1:** Characteristics of the included studies CD, Crohn’s disease; JAK, Janus kinase; OLE, open-label extension; TNF, tumor necrosis factor

Author	Year	Country	Study design	Sample size	Patient population	Dosage and frequency of upadacitinib	Comparator
Colombel et al. [16]	2024	Multicenter study	Post hoc analysis of pooled data from two phase 3, multicenter, double-blind, 12-week induction trials (U-EXCEL and U-EXCEED) and one maintenance trial (U-ENDURE)	1,021 patients (674 upadacitinib and 347 placebo)	Adults aged 18-75 years with moderately to severely active CD for ≥3 months	Upadacitinib 45 mg once daily	Placebo
Elford et al. [19]	2024	United Kingdom	Retrospective, multicenter cohort study	93 patients	Median age: 36 years (IQR 26-49); Male: 55%; Median disease duration: 12 years (IQR 6-16); Prior therapy exposure: anti-TNF: 98%, IL-12/23 or IL-23 inhibitors: 76%, anti-integrin: 53%, JAK inhibitor: 12%; 82% had exposure to at least two classes of advanced therapies, and 52% had exposure to at least three classes of advanced therapies	Upadacitinib induction: 45 mg daily for 99% of patients; Maintenance: 78% on 30 mg daily, 22% on 15 mg daily	None (single-arm study)
Peyrin-Biroulet et al. [17]	2024	Multicenter study	Post hoc analysis of pooled data from two phase 3, multicenter, double-blind, 12-week induction trials (U-EXCEL and U-EXCEED) and one maintenance trial (U-ENDURE)	1,021 patients (288 without prior biologic failure, 733 with prior biologic failure)	Moderately to severely active CD patients aged 18-75 years, with or without prior biologic failure	Upadacitinib 45 mg daily during the induction phase; maintenance with placebo, upadacitinib 15 mg, or upadacitinib 30 mg daily	Placebo
Loftus et al. [18]	2023	Multicenter study	Post hoc analysis of pooled data from two phase 3, multicenter, double-blind, 12-week induction trials (U-EXCEL and U-EXCEED) and one maintenance trial (U-ENDURE)	1,021 patients (674 upadacitinib, 347 placebo)	Adults aged 18-75 years with moderately to severely active CD for ≥3 months	Induction: 45 mg once daily for 12 weeks; Maintenance: 15 mg or 30 mg once daily for 52 weeks	Placebo
Friedberg et al. [20]	2023	United States	Prospective cohort study	40 CD patients	Adults with CD previously treated with biologics	45 mg daily for 8 weeks (82% of CD patients); 15 mg daily for others	None (single-arm study)
D'Haens et al. [21]	2022	Multicenter study	Phase II, multicenter, OLE study	107 patients	Adults with moderate-to-severe CD who completed the 52-week CELEST study	Extended-release formulation: 15 mg once daily (QD) or 30 mg QD	None (OLE)
Sandborn et al. [22]	2020	Multicenter study	Randomized, double-blind, placebo-controlled, dose-ranging phase 2 study	220 patients randomized	Adults (aged 18-75 years) with moderate to severe CD, inadequate response/intolerance to immunosuppressants or TNF antagonists	Induction: 3 mg, 6 mg, 12 mg, or 24 mg twice daily (BID), or 24 mg once daily (QD) for 16 weeks; Maintenance: 3 mg, 6 mg, or 12 mg BID, or 24 mg QD for 36 weeks	Placebo (for induction period only)

The main findings of the included studies are summarized in Table 2.

**Table 2:** Summary of the main findings of included studies AE, adverse event; APS, abdominal pain score; CD, Crohn’s disease; CDAI, Crohn’s Disease Activity Index; HBI, Harvey-Bradshaw Index; IBDQ, Inflammatory Bowel Disease Questionnaire; JAK, Janus kinase; SAE, serious adverse event; SF, stool frequency; TNF, tumor necrosis factor; UC, ulcerative colitis

Author	Outcome measures	Efficacy outcomes	Adverse events	Conclusions
Colombel et al. [16]	Daily SF, APS, SF/APS clinical remission, CDAI clinical remission, SF/APS clinical response, CR-100	SF/APS clinical remission achieved earlier with upadacitinib (median 13 days) vs. placebo (median 32 days); Higher rates of SF/APS clinical remission, CDAI clinical remission, SF/APS clinical response, and CR-100 with upadacitinib vs. placebo from week 2 through week 12. Rapid symptom relief (SF/APS clinical remission) observed within five to six days of upadacitinib treatment	Not reported in this post hoc analysis	Upadacitinib 45 mg once daily provided rapid relief of CD symptoms within the first week of treatment and improved clinical outcomes starting at week 2, regardless of prior biologic exposure
Elford et al. [19]	Treatment persistence: Week 12: 87.1%, Week 24: 81.7%, Week 52: 62.8%	Clinical remission rates: Week 12: 64% (42/66), Week 24: 48% (22/46), Week 52: 38% (8/21); CRP remission rates: Week 12: 55% (40/73), Week 24: 38% (20/53), Week 52: 19% (6/22); Fecal calprotectin remission rates: Week 12: 50% (24/48), Week 24: 36% (15/42), Week 52: 19% (3/16)	Total adverse events: 40% (37/93); Serious adverse events: 12% (11/93); Most common adverse event: Infection (15%); Adverse events causing permanent medication cessation: 10% (9/93)	Upadacitinib was effective in a real-world, highly medically refractory CD cohort with good persistence. No new safety signals were observed.
Peyrin-Biroulet et al. [17]	Clinical remission, endoscopic response, normalization of inflammation markers (CRP and fecal calprotectin), safety (adverse events)	Upadacitinib significantly outperformed placebo in clinical remission, endoscopic response, endoscopic remission, and CRP normalization at both 12 and 52 weeks, regardless of prior biologic failure status	Similar rates of adverse events between upadacitinib and placebo groups; rare serious infections, herpes zoster, neutropenia, and malignancies	Upadacitinib showed improved clinical and endoscopic outcomes in patients with CD, regardless of biologic treatment history
Loftus et al. [18]	Primary: Clinical remission (CDAI score <150) and endoscopic response (decrease in SES-CD >50% from baseline) at week 12 (induction) and week 52 (maintenance)	Induction (45 mg vs. placebo): Clinical remission: U-EXCEL 49.5% vs. 29.1%, U-EXCEED 38.9% vs. 21.1%; Endoscopic response: U-EXCEL 45.5% vs. 13.1%, U-EXCEED 34.6% vs. 3.5% (all p < 0.001). Maintenance (15 mg, 30 mg vs. placebo): Clinical remission: 37.3%, 47.6% vs. 15.1%; Endoscopic response: 27.6%, 40.1% vs. 7.3% (all p < 0.001)	Induction: Higher rates of herpes zoster, neutropenia, and creatine kinase elevation with upadacitinib. Maintenance: Dose-dependent increases in herpes zoster, hepatic disorders, neutropenia, and creatine kinase elevation	Upadacitinib was superior to placebo for induction and maintenance of clinical remission and endoscopic response in patients with moderate-to-severe CD, regardless of previous failure of biologic therapy
Friedberg et al. [20]	Clinical response and remission (HBI for CD), CRP, fecal calprotectin	76.5% clinical response, 70.6% clinical remission at week 8	32.4% experienced adverse events. Most common: acne (22.9%). Six patients discontinued due to AEs; one SAE (hospitalization for anemia)	Upadacitinib demonstrated rapid efficacy and acceptable safety in medically resistant UC and CD patients, including those previously exposed to tofacitinib
D'Haens et al. [21]	Clinical remission, enhanced clinical response, CDAI remission, endoscopic remission, endoscopic response, steroid-free remission, IBDQ remission, hsCRP and FCP levels, safety outcomes	Clinical remission 2.8/1.0 maintained at 30 months: 61% (15 mg), 54% (30 mg), 55% (dose-escalated); Enhanced clinical response at 30 months: 85% (15 mg), 74% (30 mg), 70% (dose-escalated); Endoscopic remission at 24 months: 34% (15 mg), 43% (30 mg), 0% (dose-escalated); Sustained or improved hsCRP and FCP levels across all groups	Overall AEs: 89.7% of patients (374.6 events/100 patient-years); Serious AEs: 18.7% of patients (15.3 events/100 patient-years); Most common AEs: infections (67.3% of patients); Herpes zoster: 4.7% of patients (3.1 events/100 patient-years); No new safety signals identified	Long-term treatment with upadacitinib led to sustained clinical and endoscopic improvements decreased inflammation markers, and increased patient-reported quality of life benefits in patients with CD who were mostly refractory to TNF therapy. The safety profile was consistent with previous upadacitinib studies and that of other JAK inhibitors
Sandborn et al. [22]	Primary: Clinical remission at week 16, Endoscopic remission at week 12/16; Secondary: Clinical response, Endoscopic response, CDAI <150, Corticosteroid-free remission, Changes in biomarkers (hs-CRP, fecal calprotectin), Quality of life (IBDQ)	Endoscopic remission at week 12/16: significant dose-response relationship, highest with 24 mg BID (22%, p < 0.01 vs. placebo); Clinical remission at week 16: highest with 6 mg BID (27%, p < 0.1 vs. placebo); Maintenance: 12 mg BID showed highest rates of clinical and endoscopic endpoints at week 52 (not statistically significant); Significant improvements in quality of life (IBDQ) with upadacitinib vs. placebo	Higher incidence of AEs at doses >12 mg BID; Most common AEs: headache, worsening CD, abdominal pain, fatigue, upper respiratory tract infection, urinary tract infection, nausea, vomiting, acne; Serious infections, herpes zoster, and lipid elevations were observed; Two intestinal perforations during induction (both with 24 mg doses); No deaths occurred	Upadacitinib was superior to placebo in inducing endoscopic improvements in patients with moderate to severe CD refractory to biologics. Maintenance therapy led to sustained clinical, endoscopic, and patient-reported benefits. Further evaluation in phase 3 trials is warranted

Discussion

This systematic review synthesized evidence from seven key studies evaluating the efficacy and safety of upadacitinib in CD. The findings consistently demonstrate that upadacitinib is an effective treatment for inducing and maintaining remission in patients with moderate to severe CD, including those who have failed previous biologic therapies. Key findings indicate a rapid onset of action, with Colombel et al. reporting that upadacitinib 45 mg once daily provided significant relief of CD symptoms within the first week, achieving clinical remission in a median of 13 days compared to 32 days for placebo [16]. Across multiple studies, upadacitinib showed superior efficacy compared to placebo in inducing clinical remission, with rates of 49.5% vs. 29.1% (U-EXCEL) and 38.9% vs. 21.1% (U-EXCEED) at 12 weeks, as reported by Loftus et al. [18]. Significant improvements in endoscopic outcomes were also noted, with endoscopic response rates of 45.5% vs. 13.1% (U-EXCEL) and 34.6% vs. 3.5% (U-EXCEED) at 12 weeks. Long-term efficacy was confirmed in maintenance studies, with clinical remission rates at 52 weeks of 37.3% (15 mg), 47.6% (30 mg), and 15.1% (placebo), and endoscopic response rates of 27.6% (15 mg), 40.1% (30 mg), and 7.3% (placebo). Furthermore, upadacitinib was found effective in biologic-refractory patients, as shown by Peyrin-Biroulet et al., addressing a crucial unmet need in CD management [17]. Real-world data from Elford et al. demonstrated its effectiveness in highly treatment-refractory cohorts, with clinical remission rates of 64% at week 12 and 38% at week 52 [19]. Long-term data from D'Haens et al. indicated sustained clinical and endoscopic improvements over 30 months, with 61% of patients on 15 mg and 54% on 30 mg maintaining clinical remission [21].

While direct comparative studies with other CD treatments were not included in this review, upadacitinib’s efficacy can be contextualized within the current treatment landscape. Clinical remission rates observed with upadacitinib are comparable to, or potentially higher than, those reported for anti-TNF therapies (30-50% at week 52) and anti-integrin therapies (30-40% at week 52) in their respective pivotal trials [19]. The efficacy of upadacitinib also appears to align with or surpass that reported for tofacitinib in UC, although direct comparisons in CD are not available [23]. Upadacitinib’s performance in patients who had failed conventional therapies suggests it could be a valuable option for those refractory to immunomodulators and corticosteroids. Additionally, the rapid symptom relief observed with upadacitinib (within one to two weeks) favorably contrasts with the slower onset of some biologic therapies and conventional immunomodulators [16].

While upadacitinib demonstrated an acceptable safety profile, several concerns emerged. Increased rates of infections, particularly herpes zoster, were noted, consistent with the risk profile of JAK inhibitors, necessitating vigilance and potential prophylactic measures for high-risk patients [17,18,21,22]. Dose-dependent increases in neutropenia were reported, underscoring the importance of regular blood count monitoring [17,18]. Some studies noted elevations in blood lipid levels, a class effect of JAK inhibitors, highlighting the need for lipid monitoring and management [22]. Increases in creatine kinase levels were observed, though the clinical significance remains unclear and requires further investigation [18]. Rare but serious adverse events, such as intestinal perforations, were reported, especially at higher doses, emphasizing careful patient selection and monitoring [22]. While D'Haens et al. provided data for up to 30 months, further long-term safety data is necessary to fully characterize upadacitinib's risk profile, particularly concerning malignancy and cardiovascular outcomes [21].

The findings of this review are tempered by several limitations. There was notable heterogeneity among the included studies in terms of design, patient populations, and outcome definitions, complicating direct comparisons. The absence of head-to-head trials comparing upadacitinib with other CD treatments limits the ability to definitively position it within the treatment algorithm. Potential publication bias is a concern, as negative studies may be underrepresented. Although some real-world data were included, the majority of evidence comes from controlled clinical trials, which may not fully reflect effectiveness and safety in routine practice. Incomplete long-term safety data, particularly regarding rare events and malignancy risk, leaves gaps in understanding upadacitinib’s full risk profile in CD. Additionally, data on the efficacy and safety of upadacitinib in specific populations, such as the elderly, those with comorbidities, or diverse ethnic groups, remain limited.

Future directions

The findings of this systematic review underscore several key areas for future research on upadacitinib in CD. First, long-term safety and efficacy studies with extended follow-up periods (five to 10 years) are essential to fully assess the long-term safety profile of upadacitinib, particularly concerning malignancy risk, cardiovascular outcomes, and sustained efficacy. Comparative effectiveness trials are also needed, with head-to-head RCTs comparing upadacitinib to established CD treatments like anti-TNF agents, anti-integrin therapies, and other small molecules to refine treatment algorithms and guide clinical decision-making. Additionally, combination therapy studies exploring upadacitinib with biologics or immunomodulators could enhance efficacy and should be investigated through robust clinical trials. Identifying predictive biomarkers would facilitate personalized treatment approaches, while dose optimization studies could help tailor dosing strategies to individual patient needs, balancing efficacy and safety. Larger, prospective real-world effectiveness studies across diverse settings and populations would provide important insights into upadacitinib’s performance in routine clinical practice. Furthermore, research in special populations, such as the elderly, pediatric patients, pregnant women, and those with comorbidities, is critical to establish the safety and efficacy of upadacitinib in these groups. Basic and translational research into the mechanisms of action of upadacitinib could inform future drug development and combination strategies, while more comprehensive assessments of quality of life and patient-reported outcomes would shed light on its impact on daily living. Economic evaluations comparing upadacitinib with existing treatments would also be crucial for healthcare policy and access decisions.

Given the promising results in CD, investigating the potential of upadacitinib in other inflammatory conditions is warranted. In UC, further studies could clarify its role in management, while exploring its efficacy in spondyloarthropathies, such as ankylosing spondylitis or psoriatic arthritis, especially in patients with concomitant CD, which could prove valuable. Additionally, upadacitinib’s efficacy in other immune-mediated inflammatory diseases, like atopic dermatitis or alopecia areata, where JAK-STAT signaling is involved, could expand its therapeutic applications. Lastly, studies focusing on the effect of upadacitinib on extraintestinal manifestations of CD, such as arthritis or uveitis, would provide a more comprehensive understanding of its therapeutic potential.

## Conclusions

This systematic review highlights the efficacy and safety of upadacitinib in treating moderate to severe CD. Upadacitinib shows superior efficacy compared to placebo in inducing and maintaining clinical remission and endoscopic improvement, including in biologic-refractory patients. Its rapid onset of action, with symptom relief within the first week, offers a significant advantage over some existing therapies. Long-term data suggest sustained efficacy and a generally acceptable safety profile for up to 30 months, with infections and laboratory abnormalities being the main concerns. Real-world evidence supports its effectiveness in treatment-refractory patients, addressing a critical unmet need. Upadacitinib’s oral administration and unique mechanism of action make it a valuable addition to the CD treatment landscape. However, its optimal positioning within the treatment algorithm, potential for combination therapy, and long-term safety require further investigation. Overall, upadacitinib represents a promising option that could significantly impact CD management.
